# Modelling and optimization of Ge/GaAs uni-travelling carrier photodiodes

**DOI:** 10.1038/s41598-025-93960-z

**Published:** 2025-03-15

**Authors:** Yutong Zhang, Mengxun Bai, Hui Jia, Haotian Zeng, Yangqian Wang, Huiwen Deng, Mingchu Tang, Siming Chen, Alwyn Seeds, Huiyun Liu

**Affiliations:** Department of Electronic and Electrical Engineering, University College London, Torrington Place, London, WC1E 7JE UK

**Keywords:** Optoelectronic devices and components, Optical physics, Materials science

## Abstract

Uni-Travelling-Carrier Photodiodes (UTC-PDs) are pivotal for the advancement of high-speed optical communication systems. Current UTC-PDs have a trade-off between high performance and low production costs. The performance of conventional Ge/Si UTC-PDs is limited by the materials, primarily due to the relatively low electron mobility of Si compared with that of GaAs and InP. The UTC-PDs based on high-mobility materials such as InP have high production and fabrication costs, which limits their effectiveness in next-generation applications. Germanium (Ge) and Gallium Arsenide (GaAs), with their high carrier mobility and lattice-matching capabilities, are strong candidates for UTC-PDs. Their compatibility with GaAs and Si substrates allows for large-scale production and integration into silicon photonics. The Ge/GaAs UTC-PDs are poised to meet the increasing performance demands while keeping production at relatively low costs. This study provides an in-depth analysis and simulation of Ge/GaAs-based UTC-PDs operating at 1550 nm and demonstrates an innovative front-illuminated design. Simulations indicate a 3-dB bandwidth from 30 GHz of up to 54 GHz, responsivity ranging from 0.5 A/W to 0.7 A/W at -2 V, and device diameter between 5 μm and 8 μm. This research opens avenues for a new generation of UTC-PD designs, highlighting the feasibility and potential of the Ge/GaAs material system and providing new options for the development of future photodetectors.

**Supplementary Information:**

The online version contains supplementary material available at 10.1038/s41598-025-93960-z.

## Introduction

Photodetectors are vital in optical communication systems and are widely used in a variety of bands, especially C-band 1550 nm^1,2^. Compared to traditional photodetectors, Uni-Travelling-Carrier Photodiodes (UTC-PDs) offer superior performance with higher speed and bandwidth because UTC-PDs use electrons as uni-travelling carriers, which reduces carrier recombination and transit time^3^. This advantage makes UTC-PDs highly suitable for high-frequency applications technologies^4–8^. UTC-PD involves two primary regions like ordinary photodetectors: the absorption area and the collection area​^3,9^​. The difference is that the UTC-PD design ensures only electrons are transported^3^. The materials in the absorption area require a high absorption coefficient, low bandgap, and high efficiency of carrier generation, while those in the collection area require high electron mobility and effective carrier transport. Ideally, the materials in these two areas have similar lattice constants to keep low defect density, which would be more promising for devices with better performance^9,10^.

Ge/Si has been well investigated as the absorption and collection layers for UTC-PD thanks to their lattice matching capability^11,12^. However, its 3-dB bandwidth performance is suboptimal due to the limitation of material mobility, with studies showing a maximum bandwidth of approximately below 40 GHz and relatively low responsivity, limiting the applications^11,12^. Conversely, InGaAs/InP UTC-PDs offer higher electron mobility and faster response times, making them more suitable for high-frequency applications^13–18^. Contemporary UTC-PDs exhibit impressive metrics, with responsivity over 0.1 A/W, 3-dB bandwidths over 110 GHz, and low dark currents under 100 pA^14^, which area between 14 µm^2^ to 30 µm^2^. However, InP-based devices are costly and limited by the maximum wafer size compared with Si-based devices^19^. This cost difference implies that mass-producing InGaAs/InP UTC-PDs could be much more expensive than Si-based UTC-PDs, necessitating a trade-off between price and performance^19^. Additionally, the Ge/GaAs material system shows excellent potential for UTC-PD device design^20^. Specifically, Ge has high carrier mobility and excellent infrared sensitivity with a narrow bandgap of 0.7 eV, making it ideal for absorbing near-infrared light and therefore crucial for fiber-optic communications^21^. Similar to the Ge/Si combination, Ge is qualified as a candidate for being a UTC-PD absorption region^11^. On the other hand, GaAs features a direct bandgap of 1.42 eV and high electron mobility, enabling superior performance in high-frequency and high-speed electronics, which makes it a candidate for the collection area^22,23^. More importantly, GaAs only have a 0.08% mismatch with Ge^24^, allowing for high-quality heterostructures essential for UTC-PDs. In addition, the most attractive advantages of Ge/GaAs-based UTC-PDs are lower material costs^19^ and the ability to be grown on GaAs or Si substrates^25,26^, leveraging the scalability and cost-effectiveness of silicon wafers.

Recent research has explored Ge/GaAs materials for UTC-PDs, but their current performance, particularly the 3-dB bandwidth (lower than 10 GHz) cannot meet the requirement maybe because of their considerable thickness and relatively straightforward structural configuration in comparison to other UTC-PDs^27–29^, remains inadequate for applications^20^. This study investigates the theoretical performance of Ge/GaAs UTC-PDs. It demonstrates a front-illuminated photodetector design with distributed Bragg reflector (DBR) mirrors at the bottom. From the simulation results, the UTC-PD based on this design can be applied to high frequency area and is close to InGaAs/InP UTC-PDs in performance^20^. We first calculate the band diagram and electric field diagram to verify the feasibility of the design. Then, optoelectrical characterizations including dark/photon current, responsivity, and 3-dB are given. In addition, theoretical analysis also supports the feasibility of varying the design acrossing different sizes and bias voltages, highlighting its potential applications. Based on the theoretical simulation, Ge/GaAs-based UTC-PDs achieve 3-dB bandwidth up to 54 GHz, responsivity of 0.5 A/W, and dark current of 117 nA in 5 µm diameter at an operating wavelength of 1550 nm. For front-illuminated designs, a 5 µm diameter is about the minimum practical size for normal incidence devices, so we also simulated devices with a diameter of 5 µm to 8 µm. The results mean that this design exhibits significant economic advantages with accepted performance. This research lays a foundation for applying the Ge/GaAs system in optoelectronic devices, including UTC-PDs and other photodetectors such as Avalanche Photodiodes (APDs) and modulators. Furthermore, this study guides future wafer growth and device fabrication.

## Structure and device design

The UTC-PD in this study contains a highly p-type doped Ge absorption layer and an n-type wide-bandgap GaAs collection layer. In the Ge layer, incident light generates electron-hole pairs, with holes being the majority carriers and quickly dissipating due to short dielectric relaxation times. Minority carrier electrons are directed toward the GaAs layer^30^, creating a unidirectional carrier flow. In the GaAs layer, electrons travel rapidly (2 × 10^7^ cm/s) due to the electric field at the heterojunction interface, with the UTC-PD’s response speed primarily determined by the electron transit time in the Ge and GaAs layers^31^.

**Fig. 1:**
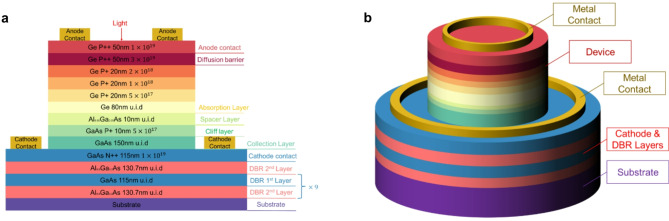
Schematic view of the reported Ge/GaAs UTC-PD design. (**a**) A cross-sectional schematic view of Ge/GaAs UTC-PD which illustrates the thickness, doping level and material of each layer. (**b**) 3D schematic view of Ge/GaAs UTC-PD.

Figure 1a illustrates the cross-sectional schematic view of the Ge/GaAs UTC-PD. The device starts with an anode contact composed of a 50 nm heavily p-type doped Ge of 1 × 10^19^ cm^− 3^. Beneath the anode contact, a diffusion barrier layer (Ge, 3 × 10^19^ cm^− 3^, 50 nm) is designed to prevent undesirable carrier diffusion, which is imperative for UTC-PDs^3,13^. The absorption layer consists of multiple sub-layers of p-type Ge with graded doping concentrations: 2 × 10^18^ cm^− 3^ (20 nm), 1 × 10^18^ cm^− 3^ (20 nm), and 5 × 10^17^ cm^− 3^ (20 nm), followed by an undoped Ge layer (80 nm). This grading creates a quasi-electric field aiding in carrier transport^32^. The incorporation of a 10 nm undoped Al_0.05_Ga_0.95_As spacer layer at the Ge/GaAs heterojunction effectively mitigates bandgap discontinuities, ensuring seamless electron transport. The addition of spacer layers to reduce band discontinuities is a well-established approach in heterojunction design in previous studies^33–35^. The 5% Al content is specifically chosen to balance conduction band alignment. Higher compositions would introduce an well, hindering carrier injection, while lower compositions would fail to sufficiently reduce the discontinuity. Thermionic emission is an inherent characteristic of heterojunctions. However, its impact at this interface is significantly reduced due to the incorporation of 5% Al, which optimizes the potential barrier and smooths the band structure. This reduction ensures that its contribution to carrier loss is negligible, allowing it to be effectively ignored in this design. Subsequently, a 10 nm n doped GaAs cliff layer (1 × 10^18^ cm^− 3^) enhances the electric field adjacent to the absorption layer, facilitating efficient carrier transport. It can be observed that the design details of this layer are also referenced in past research^3,36^. The collection layer comprises a 150 nm undoped GaAs layer to ensure rapid electron collection. Below the collection layer is n-type doped GaAs (1 × 10^19^ cm^− 3^, 115 nm thick), which acts as a cathode contact and forms the first of the ten pairs of DBRs with the undoped Al_0.8_Ga_0.2_As (130.7 nm thick) below. The DBR composed of GaAs 115 nm/ Al_0.8_Ga_0.2_As 130.7 nm can achieve very high reflectivity at 1550 nm^37^, reflecting the light penetrating from the absorption region to reach the absorption layer again to form secondary absorption. Photodetectors with DBR mirrors have been shown to have a significant effect on improving photocurrent and responsivity by reflection light, without impact of the electrons transport^38^. Furthermore, this device structure is supported by a buffer layer to accommodate lattice mismatch, built on a silicon substrate providing mechanical stability.

Photodiodes are classified into front-illumination, back-illumination, and planar designs, each with specific characteristics. Front-illumination photodiodes allow light to directly enter the absorption layer through the top surface, simplifying fabrication and integrating effectively with DBR structures to enhance responsivity. Back-illumination photodiodes, where light enters through the substrate, free the top surface for advanced circuitry and improve waveguide coupling but require complex fabrication and transparent substrates. Planar photodiodes, commonly used in waveguide designs, excel in compactness and high-speed performance due to low capacitance but often suffer from limited optical absorption in the active region. The front-illumination design is selected for the Ge/GaAs UTC-PD due to its straightforward design and fabrication process, its ability to provide accurate evaluation of absorption efficiency in simulations, and its seamless compatibility with DBR structures, which enhances light trapping and overall responsivity. Based on these characteristics, we chose the front-illumination design as the starting point for our research. The 3D schematic view is shown in Fig. 1b.

The dimensions of the UTC-PD mentioned in this work all refer to the upper half (above the cathode layer in blue). Compared with the UTC-PD planar design with a waveguide, we adopt a circular front-illuminated design with DBR. The cylindrical design with circular electrodes in UTC-PDs enhances light absorption and electrical performance by ensuring uniform light distribution and reducing series resistance, which causes higher responsivity and photocurrent amplitude. We further amplify this advantage by introducing the DBR structure. However, cylindrical photodetectors are limited in size due to light coupling loss, so the minimum diameter of our design is limited to 5 µm, which will affect the upper bound of the bandwidth.

In this design, the anode and cathode electrodes are optimized for efficient optical coupling and current collection. The anode diameter is kept near the lower limit for effective light absorption, while the cathode contact layer is slightly larger (roughly around 2–3 µm) to facilitate better carrier collection and maintain low resistance^39,40^. These dimensions are based on simulation data and are expected to provide optimal performance under ideal conditions. However, actual fabrication parameters may be adjusted to account for process variations and material tolerances, ensuring the final device maintains high responsivity and stable operation. Compared with the Ge/Si UTC-PD also in front-illuminated, our design has significant performance advantages. The specific comparison will be introduced in Table 2. In future work, the Ge/GaAs UTC-PD with waveguide design can be considered to enhance the device performance and further compare it with the InGaAs/InP UTC-PD with waveguide design.

Based on the presented design, simulations are conducted using Ansys Lumerical Finite Difference Time Domain (FDTD) and CHARGE software. The version and URL are provided in the supplementary materials. Firstly, the output of the optical generation rate is obtained by defining the geometric structure of the device, the material properties and the input optical source in FDTD. The optical generation rate is then fed into the CHARGE module with the defined metal contact and doping level to obtain the photocurrent, dark current, responsivity and 3-dB bandwidth for the device. In addition, high-field mobility simulations for GaAs, AlGaAs, and Ge materials are enabled to produce more accurate results.

## Simulation results

### Working mechanism of UTC-PD

**Fig. 2:**
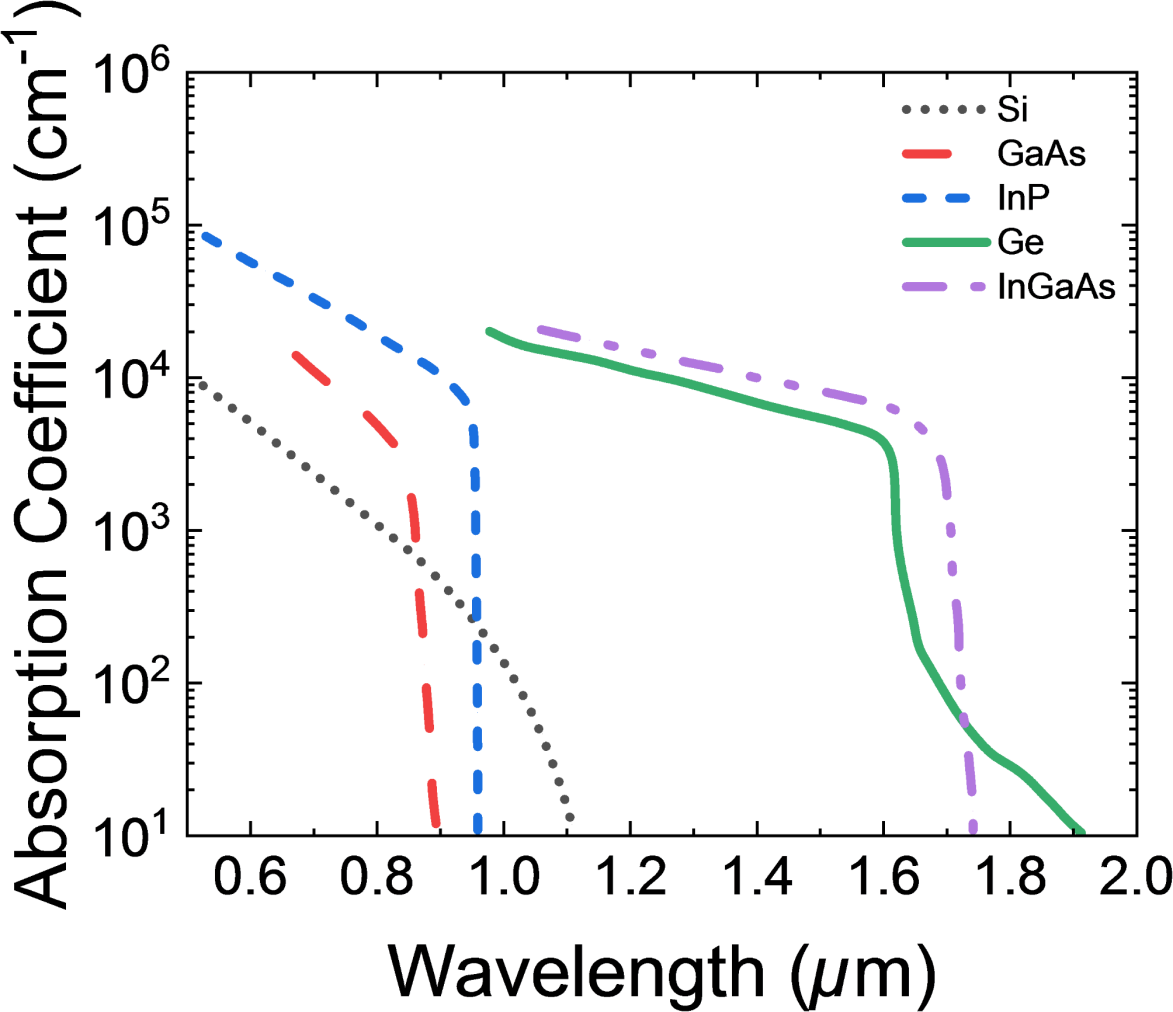
Absorption spectra for different materials.

The selection of Ge as the absorption layer and GaAs as the collection layer is further supported by their respective absorption and transport characteristics, as shown in Fig. 2. Although Ge demonstrates high absorption efficiency around 0.9 µm, it still maintains relatively good absorption at 1.55 µm, making it suitable for absorption layer of UTC-PD. It has been widely adopted in photodetector designs due to its cost-effectiveness and sufficient absorption efficiency within the 1.3–1.55 µm range^11,20,41^. GaAs, with negligible absorption beyond 1.1 µm, ensures efficient carrier transport without parasitic losses, further highlighting its role as an ideal collection layer in the proposed structure.

**Fig. 3:**
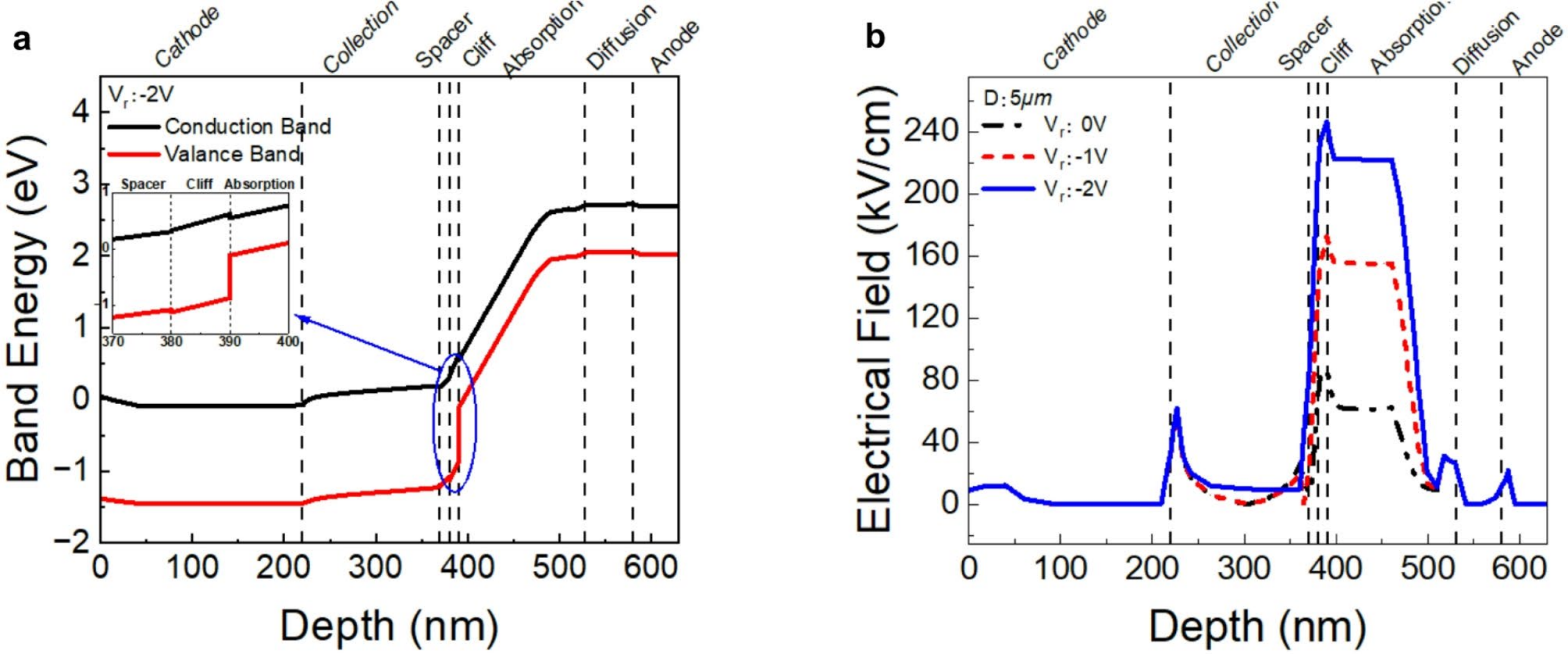
Band diagram and Electric Field Diagram for diameter with 5 µm Ge/GaAs UTC-PD. (**a**) The band diagram of the Ge/GaAs UTC-PD illustrates the conduction band (black) and valence band (red) energies as a function of depth. (**b**) The electric field diagram shows the electric field intensity as a function of depth within the Ge/GaAs UTC-PD at various biases (0 V, -1 V, -2 V). Both diagrams demonstrate the various layers, including the cathode contact, collection layer, spacer layer, cliff layer, absorption layer, diffusion barrier, and anode contact.

The working mechanism of the Ge/GaAs UTC-PD is illustrated in Fig. 3. The figures show the trend of band energy changes and the distribution of electric field strength from the anode contact layer to the cathode contact layer. The band diagram in Fig. 3a illustrates the conduction and valence band energies as a function of depth within the Ge/GaAs UTC-PD. From the cathode contact, the conduction band remains relatively flat. Besides, it can be seen that the conduction band of the collection layer is designed to have a slope to accelerate the carrier (quasi-ballistic transport). The potential remains stable until the interface with the spacer layer, where a slight discontinuity can be observed. This discontinuity, associated with the Al_0.05_Ga_0.95_As spacer layer, is designed to reduce bandgap differences and maintain a 220–240 KV/cm electric field across the absorption layer, thus enhancing carrier transport^28^. The band diagram reveals the presence of the cliff layer, which smooths the transition between the absorption and collection layers. This smoothing effect is crucial for reducing potential barriers that could impede carrier flow. Besides, the cliff layer with a 1 × 10^18^ cm^− 3^ doping level can help electrons cross the heterogeneous interfacial barriers and mitigate the effect of space charge at a high current level^34^. The absorption layer, comprising graded p-type Ge, exhibits a considerable enhancement in the conduction band energy, thereby creating a quasi-electric field that facilitates the efficient separation and transport of photo-generated carriers^3,11^. Light is absorbed in the absorption layer to produce electron-hole pairs; due to the concentration difference, electrons and holes will be diffused to both ends of the absorber layer simultaneously. Finally, the diffusion barrier near the anode contact maintains the electric field, preventing the backflow of carriers and ensuring unidirectional carrier transport towards the collection region.

The electric field diagram in Fig. 3b illustrates the electric field intensity as a function of depth within the 2 µm diameter (D) Ge/GaAs UTC-PD at various applied biases (0 V, -1 V, -2 V). At 0 V bias, the electric field profile remains relatively flat across most of the device, with noticeable peaks at specific interfaces. These peaks correspond to transitions between different material layers, such as the spacer and absorption layers, indicating regions where the electric field is strongest. The presence of a high electric field in these regions is essential for efficient carrier separation and transport. As the reverse bias is increased to -1 V and further to -2 V, the electric field intensity within the device increases significantly. This increase in electric field strength enhances the separation and drift of photo-generated carriers, thereby reducing the recombination rates and improving the overall responsivity of the photodiode. Figure 3b also illustrates that the electric field peaks become more pronounced as the reverse bias increases. Specifically, the peak electric fields observed at the interface between the absorption layer and the cliff layer, as well as between the cliff layer and the collection layer, are of critical importance for the rapid collection of electrons. Additionally, the electrons will be accelerated to overshoot velocity^31,42^ while across the n-doped GaAs cliff layer with nearly 250 KV/cm electric field. The steep gradients in these regions facilitate the swift transport of carriers, which is essential for achieving high-speed performance and large bandwidth in the UTC-PD. Furthermore, the increased electric field under higher reverse biases helps to maintain a strong field across the absorption layer. This is crucial in ensuring that photo-generated carriers are efficiently swept into the collection layer, thereby minimizing transit times and enhancing the 3-dB bandwidth of the device. The energy band diagrams, and electric field diagrams of the structure exhibit a high degree of similarity to those of UTC-PDs made of other materials, which increases the credibility of the material combination.

### Photocurrent and dark current characteristics

In order to ascertain the viability of the device under a range of conditions, it is essential to demonstrate its functionality in a simulated environment. In this section, several important characteristics of Ge/GaAs UTC-PD at different operating voltages and different sizes are presented. High photocurrent and low dark current are essential for the optoelectrical performance of UTC-PDs, and both are closely linked to the device bias voltage magnitude and surface area.

**Fig. 4:**
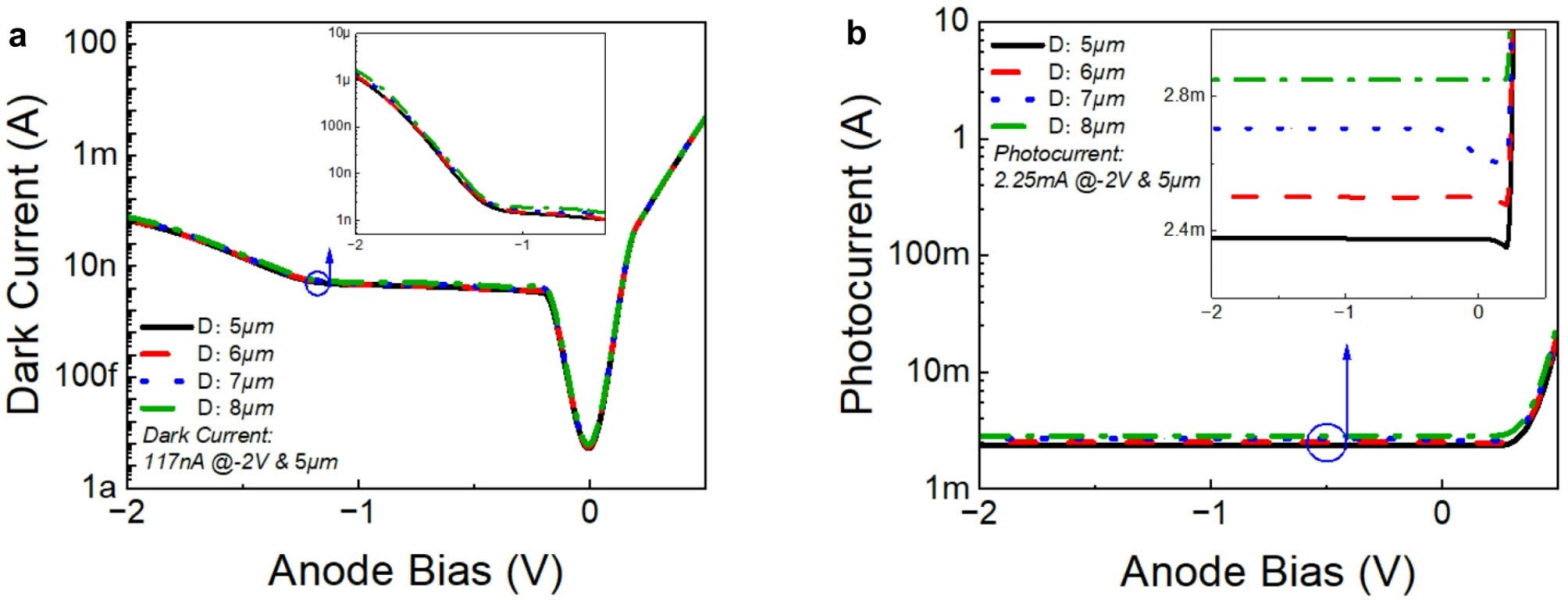
Dark currents and photocurrent from Ge/GaAs UTC-PD with different device size conditions. (**a**) Dark current versus voltage characteristics for Ge/GaAs UTC-PD devices with varying diameters (5 µm to 8 µm) at different bias conditions. (**b**) Photocurrent versus voltage characteristics for Ge/GaAs UTC-PD devices with varying diameters (5 µm to 8 µm) at different bias conditions.

Figure 4a presents the dark current characteristics of devices with diameters ranging from 5 to 8 µm, measured under different bias conditions. The figure reveals two distinct regions in the dark current response: a relatively flat region extending to a bias of -1.25 V and a pronounced increase beyond − 1.25 V. This trend is consistent with the findings of previous UTC-PD dark current measurement^14^. In the flat region, the primary contribution to the dark current is Shockley–Read–Hall (SRH) recombination, which is facilitated by trap states^43^. Conversely, in the region where the dark current increases sharply, band-to-band tunnelling becomes the dominant mechanism^14^. In addition, the dark current shows a noticeable dependence on the device diameter. For the 5 µm device, the dark current is 117 nA at -2 V bias, which is much lower than other Ge/Si UTC-PDs^11,12,41^ and its performance is comparable to that of InGaAs/InP^44,45^. With increasing diameter, the dark current values for devices from 5 µm to 8 µm approximately increase from 117 nA to 1.64 *µ*A, respectively.

Figure 4b presents the photocurrent characteristics of Ge/GaAs devices with diameters ranging from 5 to 8 μm, measured under the same bias conditions of dark current. The simulation of photocurrent for the front-illuminated photodetector must account for power absorption and the collection efficiency of photogenerated carriers. In this case, we assume a 100% carrier collection efficiency. Consequently, the absorption power density $\:{P}_{abs}$ primarily dictates the photocurrent and responsivity, as expressed by the following formula^46^:

1 $$\:\begin{array}{l}{P}_{abs}=-0.5real\left(\overrightarrow{\nabla\:}\cdot\:\overrightarrow{P}\right)=-0.5real\left(i\omega\:\overrightarrow{E}\cdot\:{\overrightarrow{D}}^{*}\right)=-0.5\omega\:{\left|E\right|}^{2}imag\left(\epsilon\:\right)\end{array}$$

It is possible to calculate the absorption directly from this formula, but divergence calculations tend to be very sensitive to numerical problems. Where $\:\overrightarrow{D}$ is electric displacement field, defined as $\:\overrightarrow{D}=\epsilon\:\overrightarrow{E}$. Fortunately, there is a more numerically stable form. Where $\:\epsilon\:$ is permittivity, $\:E$ is the electric field intensity. To calculate the absorption as a function of space and frequency, we only need to know the electric field intensity and the imaginary part of the permittivity. Both quantities are easy to measure in an FDTD simulation. The number of absorbed photons per unit volume per second $\:g$ can then be calculated by dividing this value by the energy per photon^47^:

2 $$\:\begin{array}{l}g=\frac{{P}_{abs}}{\hslash\:\omega\:}=\frac{-0.5{\left|E\right|}^{2}imag\left(\epsilon\:\right)}{\hslash\:}\end{array}$$

The absorbed photons will generate electron-hole pairs which will be separated out of the depletion region by the electric field and produce a flow of current^47^:

3 $$\:\begin{array}{l}{I}_{ph}=\frac{{P}_{abs}}{hv}\cdot\:q\end{array}$$

where $\:{P}_{abs}$ is the absorbed power per unit volume, $\:hv$ is the energy of the photon and $\:q$ is the charge of the electron (about $\:1\times\:{10}^{-19}$ coulombs).

Based on the figure, this UTC-PD operates at 0 bias or even positive bias, as the photocurrent remains almost constant from 0.4 to -2 V. Compared to the existing UTC-PD, the results align well with the characteristics. The photocurrent, dark current and PDCR(Photo-to-Dark-Current Ratio) for 5 μm device at -2 V reverse bias are 2.25 mA, 117nA and $\:1.92\:\times\:\:{10}^{4}$, respectively. According to the previous studies^48^, the PDCR for normal UTC-PD is around $\:{10}^{3}\sim{10}^{4}$, our design falls within this expected range. For a UTC-PD, the absorption and collection layers are spatially separated. Electrons, which are the minority carriers in the p-type absorption layer, diffuse towards the collection layer. This separation allows the photodiode to maintain efficient operation even under forward bias conditions, as the collection of carriers (electrons) is less affected by the forward bias that would typically lead to recombination in a conventional PIN-PD. Additionally, the graded doping profile in the absorption layer creates a quasi-electric field that aids in efficient carrier separation and transport. This field drives the minority electrons towards the collection layer, reducing the chances of recombination even when the device is forward-biased. The photocurrent also shows a noticeable dependence on the device diameter. The increasing trend in photocurrent with device diameter is expected due to the larger active area available for photon absorption, leading to higher electron-hole pair generation and, consequently, higher photocurrent^49^. For the 5 µm device at -2 V, the photocurrent is approximately 2.25 mA. With increasing diameter, the photocurrent values for devices from 5 to 8 μm are approximately 2.25 mA to 2.89 mA, respectively.

### Responsivity characteristics

In Ge/GaAs devices, the high absorption coefficient of Ge at the operational wavelength ensures efficient photon absorption. Additionally, the diffusion length of carriers in Ge is sufficient to ensure that a significant proportion of generated carriers contribute to the photocurrent.

Responsivity is another fundamental parameter that influences the sensitivity, efficiency, and signal strength of UTC-PDs. Responsivity is defined as the ratio of the photocurrent $\:{I}_{ph}$ to the incident optical power Pin, and the responsivity $\:R$ is defined as the ratio of the incident optical power to the photocurrent, which the formula can represent^50^:

4 $$\:\begin{array}{l}R=\frac{I\left(mA\right)}{P\left(mW\right)}=\eta\:\frac{q}{h\nu\:}=\eta\:\frac{q\lambda\:}{hc}\end{array}$$

where $\:I$ is the photocurrent, $\:P$ is the incident optical power, $\:q$ is the elementary charge, $\:\lambda\:$ is the wavelength of light, and $\:hc$ is Planck’s constant. In this work, responsivity is primarily calculated using this direct ratio of photocurrent to incident optical power. Alternatively, $\:R$ can also be expressed in terms of the quantum efficiency $\:\eta\:$ as shown, which represents the fraction of incident photons that generate collected carriers. Using this relationship, $\:\eta\:\:$can be derived from the simulated responsivity, making it a critical parameter for evaluating the photodiode’s ability. In this study, we mainly focus on $\:R$ as the important characteristic for photodiodes to analysis.

It is important to note that, since the light source is positioned near the device surface and its area matches that of the device, light coupling losses are not considered in the simulation. This results in a higher responsivity, but as a preliminary simulation for design guidance, this approach is acceptable. For example, in the simulation of a Ge/Si front-illuminated design^11^, the theoretical responsivity was 0.196 A/W, whereas the actual measured value was 0.18 A/W. As mentioned, simulation values are typically 1.4 to 1.6 times higher than experimental results^12^.

It is also important to mention that this study focuses on simulating the device’s external responsivity ($\:{R}_{ext}$), which accounts for the photocurrent generated relative to the total incident optical power. However, the internal responsivity ($\:{R}_{int}$), which reflects the efficiency of the absorption layer itself, is equally critical for understanding the device’s performance. $\:{R}_{int}$ is defined as the photocurrent generated per unit of optical power absorbed by the active region and can be expressed as:

5 $$\:\begin{array}{l}{R}_{int}=\frac{{R}_{ext}}{A}\end{array}$$

Where A is the absorption efficiency. By distinguishing between $\:{R}_{ext}$ and $\:{R}_{int}$, it can more accurately evaluate the performance of the absorption layer and identify opportunities to improve material or structural properties. For the Ge absorption layer, DBR mirrors is expected to boost absorption efficiency A from a baseline of 44% (without DBR)^51^ to approximately 1.5-2 times larger^52^. This improvement is achieved by reflecting unabsorbed light back into the active region, increasing the interaction length and overall absorption. Such a gain in absorption efficiency is critical for thin active layers, where light trapping is essential to optimize responsivity and quantum efficiency, particularly in high-speed photonic applications^53,54^.

**Fig. 5:**
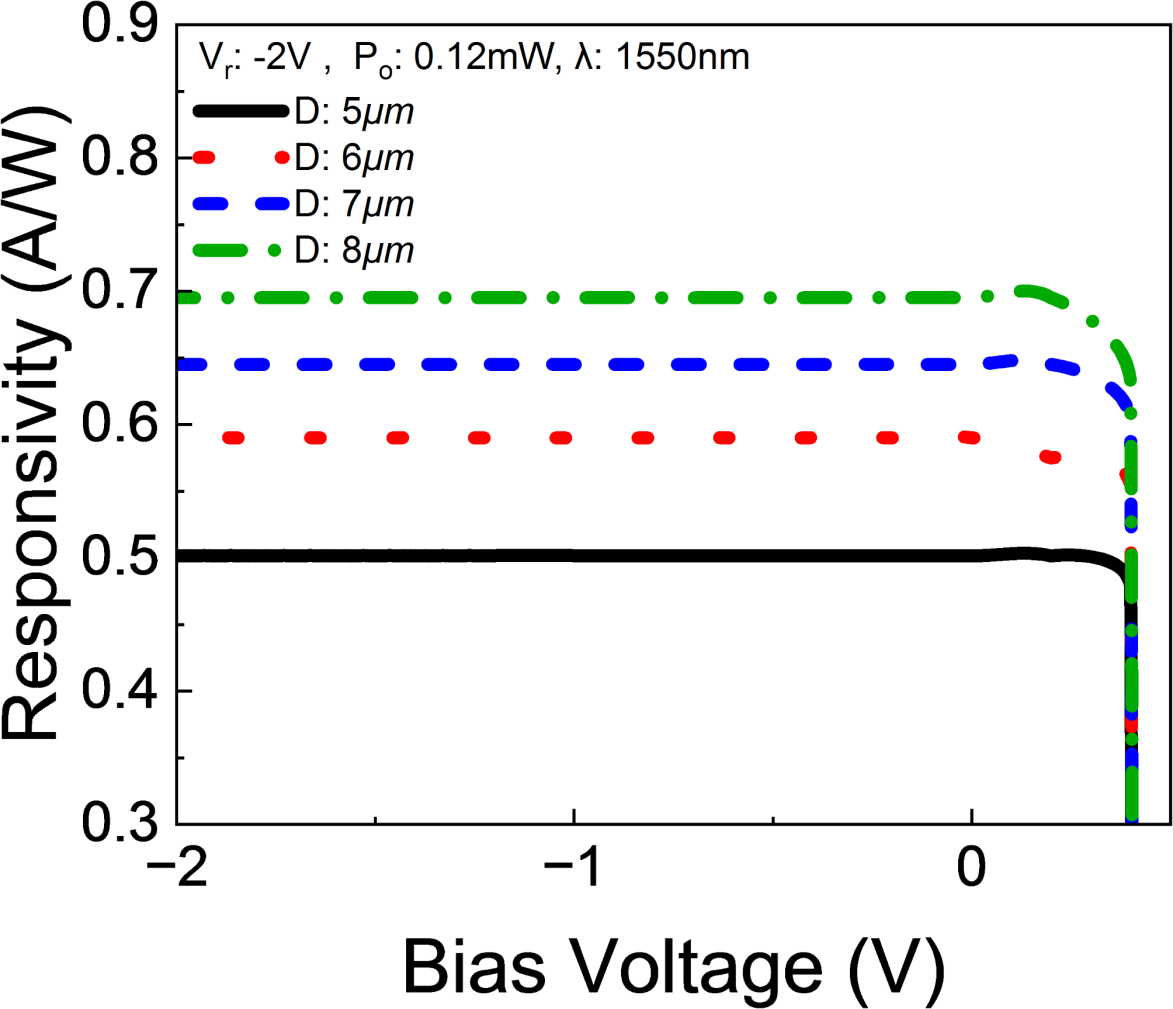
Responsivity of Ge/GaAs UTC-PD with different sizes and bias conditions. This simulation using 1550 nm wavelength optical source.

The responsivity of Ge/GaAs devices with different diameters ranging from 5 to 8 μm at a bias voltage of -2 V is shown in Fig. 5. The responsivity, measured in amperes per watt (A/W), increases from approximately 0.5 A/W for the 5 μm device to about 0.7 A/W for the 8 μm device. This trend indicates that as the device diameter increases, the responsivity also increases. The larger active area in devices with greater diameters results in more photon absorption, leading to a higher generation of electron-hole pairs and, consequently, increased photocurrent^45^. In Ge/GaAs devices, the high absorption coefficient of Ge at the operational wavelength ensures efficient photon absorption. Additionally, the diffusion length of carriers in Ge is sufficient to ensure that a significant proportion of generated carriers contribute to the photocurrent.

Following the analysis of the DBR in the previous section, we also present the impact of the DBR on the photocurrent and responsivity of devices with varying diameters under a -2 V bias voltage in Table 1.

**Table 1:** A comparison of performances for UTC-PDs with and without DBR layers working on 1550 nm.

Bias Voltage − 2 V		5 µm	6 µm	7 µm	8 µm
With DBR Layers	Photocurrent (mA)	2.25	2.39	2.62	2.89
	Responsivity (A/W)	0.5	0.53	0.59	0.7
Without DBR Layers	Photocurrent (mA)	0.89	1.01	1.08	1.12
	Responsivity (A/W)	0.176	0.193	0.235	0.248

It is evident that the incorporation of the DBR structure results in substantial improvements in both photocurrent and responsivity based on the above calculation. For instance, in the 5 μm design, the photocurrent without DBR was 0.89 mA, with a responsivity of only 0.176 A/W, which is relatively low even for UTC-PDs. However, with the DBR structure, the photocurrent increased to 2.25 mA, and the responsivity improved to 0.5 A/W. This enhancement underscores the critical role of the DBR structure, demonstrating that the Ge/GaAs UTC-PD have the ability to achieve both high 3-dB bandwidth and improved responsivity.

### Bandwidth characteristics

Good current performance establishes a UTC-PD’s baseline performance, while an excellent 3-dB bandwidth defines its upper-performance limit. This bandwidth is one of the most crucial performance metrics for photodetectors. In this simulation, small signal AC simulation is used to determine the bandwidth of the device. By applying a small sine wave signal, measuring the device’s response (output current) at different frequencies, and plotting the frequency response curve after normalization, the 3 dB frequency position is obtained. The following formula describes the frequency response of the AC input signal in the circuit:

6 $$\:\begin{array}{l}{I}_{out}\left(f\right)={I}_{in}\left(f\right)H\left(f\right)\end{array}$$

where $\:H\left(f\right)$ is the transfer function of the system and describes the output current with respect to the input current, $\:{I}_{in}\left(f\right)$ is the AC photocurrent generated by the input optical signal and $\:{I}_{out}\left(f\right)$ is the output current, which is affected by the parasitic and carrier transport effects of the device.

In the small signal model, the photodetector equivalent circuit can usually be approximated as a parallel resistor $\:R$ and capacitor $\:C$, plus the transmission time effect. The complete frequency response $\:H\left(f\right)$ is usually the result of the $\:RC$ limitation and the carrier transmission time. Its expression can be written as^55^:

7 $$\begin{array}{*{20}l} {H\left( f \right) = \frac{1}{{\sqrt {1 + \left( {{f \mathord{\left/ {\vphantom {f {f_{{RC}} }}} \right. \kern-\nulldelimiterspace} {f_{{RC}} }}} \right)^{2} } }} \cdot \frac{1}{{\sqrt {1 + + \left( {{f \mathord{\left/ {\vphantom {f {f_{T} }}} \right. \kern-\nulldelimiterspace} {f_{T} }}} \right)^{2} } }}} \\ \end{array} ~$$

8 $$\:\begin{array}{l}{f}_{RC}=\frac{1}{2\pi\:RC}\:\end{array}$$

9 $$\:\begin{array}{l}{f}_{transit}=\frac{0\cdot\:445}{{T}_{transit}}\end{array}$$

Where $\:{f}_{RC}$ is the 3 dB bandwidth can be attributed to the effects of the RC circuit and $\:{f}_{transit}$ is 3 dB bandwidth that consequence of the carrier transmission time. Total capacity $\:\left(C\right)$ is obtained by the following formula

10 $$\:\begin{array}{l}C={C}_{d}+{C}_{sub}+{C}_{c}\end{array}$$

where $\:{C}_{d}$ is the Capacitance of the Depletion or Collection Layer, $\:{C}_{sub}$ is the Substrate Capacitance, $\:{C}_{c}$ is the Contact Capacitance. All are autonomic calculated by the simulation system based on the device structure and size.

$\:R$ is obtained by the following formula

11 $$\:\begin{array}{l}R={R}_{L}+\frac{{\rho\:}_{C}}{A}\end{array}$$

$\:{R}_{L}$ is assumed to be 50 $\:\varOmega\:$, $\:{\rho\:}_{C}$ is the resistivity of metal contact^56^. The transit time $\:{T}_{transit}$ refers to the time it takes for electrons to migrate from the absorption region to the electrode, which is determined by the electric field strength and material and is also calculated by the simulation. It should be noted that $\:R$ and $\:C$ are related to the size of the device, while $\:\tau\:$ transit does not change with the size of the device but will be affected by the bias voltage.

**Fig. 6:**
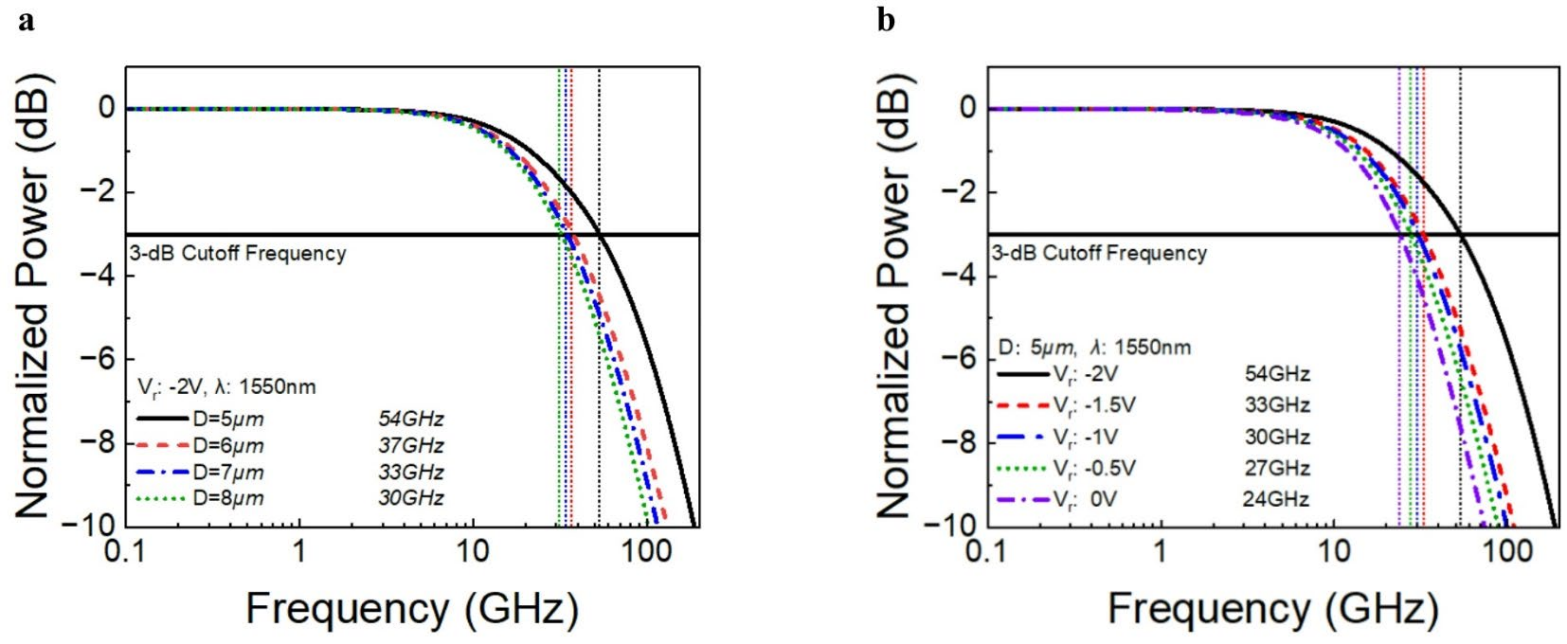
Small signal optoelectronic frequency response. Both simulations measured at a photocurrent with optical output and wavelength (*λ*) for input light source is 1550nm. (**a**) Frequency response simulation versus device size for Ge/GaAs UTC-PDs at a bias voltage of -2V. (**b**) Frequency response simulation for a 2 µm Ge/GaAs UTC-PD under different bias conditions.

Figure 6a illustrates the 3-dB bandwidth characteristics of Ge/GaAs devices with varying diameters (5 µm to 8 µm) at a fixed reverse bias voltage of -2V. The 3-dB bandwidth is observed to decrease with increasing device diameter. For the 5 µm device, the 3-dB bandwidth is approximately 54 GHz. As the diameter increases from 5 to 8 µm, the 3-dB bandwidth decreases from 54 to 30 GHz, respectively. This trend suggests that smaller device diameters result in higher bandwidths. The decrease in bandwidth with larger diameters is due to the increased capacitance and longer carrier transit times associated with larger active areas, which limit the speed of the photodetector^27^.

Figure 6b illustrates the 3-dB bandwidth characteristics of a Ge/GaAs device with a 5 µm diameter, measured at different bias voltages ranging from 0 V to -2 V. The 3-dB cutoff frequency is observed to increase with the magnitude of the reverse bias voltage. At 0 V, the 3-dB bandwidth is approximately 24 GHz. As the reverse bias increases from 0 V to -2 V, the 3-dB bandwidth increases from 24 GHz to 54 GHz, respectively. This trend indicates that applying a higher reverse bias voltage enhances the bandwidth of the device. The increase in bandwidth with higher reverse bias can be attributed to the reduction in the carrier transit time and the widening of the depletion region, which reduces the capacitance and enhances the speed of the photodetector^57^.

**Fig. 7:**
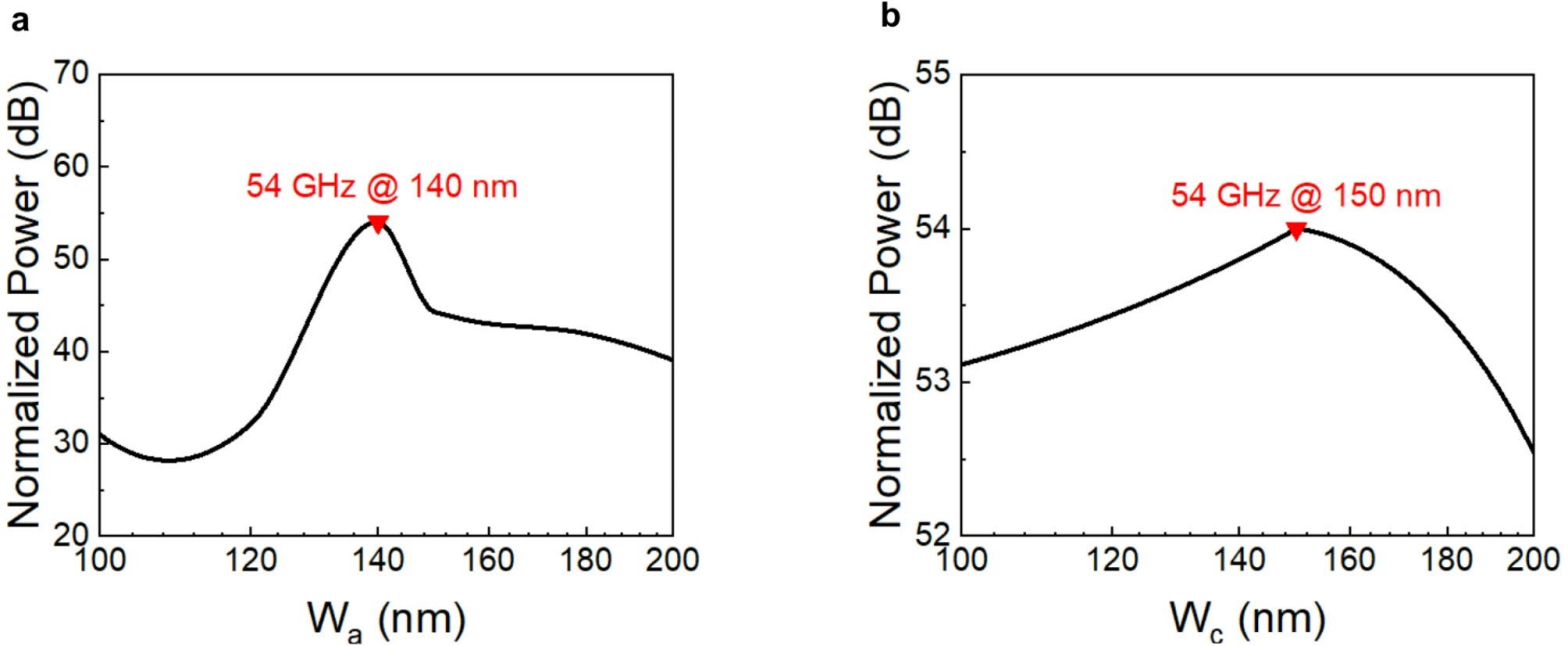
The relationship between the thickness of the absorption, collection layers and the frequency response in a Ge/GaAs UTC-PD. (**a**) This curve represents the 3-dB bandwidth as a function of the absorption layer (W_a_) thickness. (**b**) This curve represents the 3-dB bandwidth as a function of the collection layer (W_c_) thickness.

Furthermore, the thickness of the collection layer and absorption layer has been optimized to achieve the desired 3-dB bandwidth. Figure 7 illustrates how the bandwidth of the device changes when the collection and absorption layer thickness sweep from 100 to 200 nm, keeping the other layer thicknesses constant. Figure 7a illustrates the 3-dB bandwidth as a function of absorption layer thickness, while Fig. 7b shows the 3-dB bandwidth as a function of collection layer thickness. The curve in Fig. 7a peaks at a specific absorption layer thickness, indicating an optimal point where the photodiode achieves maximum bandwidth. A thinner absorption layer may not absorb enough photons, leading to lower photo-generated carrier density, while a thicker absorption layer might cause longer carrier transit times, which will cause reduced bandwidth. The chosen thickness for the absorption layer is the peak of the curve, where the bandwidth is maximized, ensuring efficient photon absorption and carrier transport. The curve for the collection layer is relatively flat, indicating that the 3-dB bandwidth is less sensitive to the thickness of the collection layer compared to the absorption layer, suggesting that variations in the collection layer thickness have a minor impact on the overall bandwidth. While its thickness does not significantly affect bandwidth, it must be sufficient to ensure efficient carrier collection and minimize transit times. Consequently, after the calculation, 150 nm and 140 nm have been finally selected as the thickness of the collection and absorption layers, respectively.

The comparison between the Ge/GaAs UTC-PDs and the existing UTC-PDs working on 1550 nm optical input is shown in Table 2. Compared to previously proposed Ge/GaAs and earlier Ge/Si UTC-PDs, our design’s responsivity is higher, and the 3-dB bandwidth is significantly better due to the optimized structural design and smaller size. In contrast, the characteristics of our Ge/GaAs devices are close to those of InGaAs/InP devices. Although its 3-dB bandwidth is still below that of the reported InGaAs/InP UTC-PD, the Ge/GaAs UTC-PD design remains highly competitive when considering the cost. In future studies, structures containing waveguides will be considered to explore the potential of 3-dB bandwidth further.

**Table 2:** A comparison of performances for this work and other UTC-PDs working on 1550 nm optical source.

3-dB bandwidth (GHz)	Photocurrent (mA)	Dark current (nA)	Size (µm)	Responsivity (A/W)	Material	Illumination type	References
54 @ -2 V	2.25 @ -2 V	117 @ -2 V	D: 5	0.5	Ge/GaAs	Vertical	This work
9.3 @ -2 V	-	8 @ -1 V	D: 10	0.11	Ge/GaAs	Vertical	^20^
12.8 @ -1.5 V	-	30,000 @ -2 V	D: 27	0.36	InP	Vertical	^33^
52.2@ -2 V	-	-	D: 14	0.33	InP	Vertical	^58^
110 @ -4 V	23 @ -4 V	50 @ -4 V	2 × 25	0.53	InP	Waveguide	^45^
9.3 @ -5 V	2.5 @ -2 V	1000 @ 0 V	D: 15	0.18	Ge/Si	Vertical	^11^
18 @ -3 V	20 @ -3 V	8900 @ -2 V	D: 14	0.12	Ge/Si	Vertical	^12^
40 @ -3 V	1.5 @ -5 V	2000 @ -5 V	4 × 13	0.5	Ge/Si	Waveguide	^59^

## Conclusion

In summary, this work presents a new design of the Ge/GaAs vertical UTC-PD with comprehensive simulation, including optoelectrical characterization and 3-dB bandwidth tests. Based on our simulations, the performance of Ge/GaAs UTC-PDs is comparable to that of InP/InGaAs UTC-PDs, with 3-dB bandwidths ranging from 30 GHz to 54 GHz and responsivity between 0.5 A/W and 0.7 A/W across device sizes from 5 µm to 8 µm. This device simulation reveals the notable potential performance of Ge/GaAs compared to InGaAs/InP UTC-PDs. Additionally, Ge/GaAs devices can be produced on larger, more cost-effective substrates, supporting mass production and broader application in silicon photonics. This study provides a foundation for the design and preparation of Ge/GaAs UTC-PDs, which are a strategic choice for both research and commercial applications, addressing the growing demand for high-speed, high-responsivity photodetectors in advanced optical communication systems.

## Electronic supplementary material

Below is the link to the electronic supplementary material.

Supplementary Material 1.

## Data Availability

The data that support the findings of this study are available from the corresponding author upon reasonable request.
