# Supplementary material for: Modelling and optimization of Ge/GaAs uni-travelling carrier photodiodes
Source: Sci Rep. 2025 Mar 15;15:8983. doi: 10.1038/s41598-025-93960-z (PMC11910542; doi:10.1038/s41598-025-93960-z)
Supplement: Supplementary file 1 — Supplementary Material 1. [file 41598_2025_93960_MOESM1_ESM.docx]

# Supplementary Materials

The simulations conducted in this study utilized ANSYS Lumerical (2024 R1).

The official URL: https://www.ansys.com/en-gb/products/optics/fdtd
